# Why Differentiation Therapy Sometimes Fails: Molecular Mechanisms of Resistance to Retinoids

**DOI:** 10.3390/ijms19010132

**Published:** 2018-01-03

**Authors:** Petr Chlapek, Viera Slavikova, Pavel Mazanek, Jaroslav Sterba, Renata Veselska

**Affiliations:** 1 Laboratory of Tumor Biology, Department of Experimental Biology, Faculty of Science, Masaryk University, 61137 Brno, Czech Republic; chlapek@sci.muni.cz (P.C.); slavikova@sci.muni.cz (V.S.); 2 International Clinical Research Center, St. Anne’s University Hospital, 65691 Brno, Czech Republic; sterba.jaroslav@fnbrno.cz; 3 Department of Pediatric Oncology, University Hospital Brno and Faculty of Medicine, Masaryk University, 62500 Brno, Czech Republic; mazanek.pavel@fnbrno.cz

**Keywords:** retinoids, cell differentiation, differentiation therapy, mechanisms of resistance

## Abstract

Retinoids represent a popular group of differentiation inducers that are successfully used in oncology for treatment of acute promyelocytic leukemia in adults and of neuroblastoma in children. The therapeutic potential of retinoids is based on their key role in the regulation of cell differentiation, growth, and apoptosis, which provides a basis for their use both in cancer therapy and chemoprevention. Nevertheless, patients treated with retinoids often exhibit or develop resistance to this therapy. Although resistance to retinoids is commonly categorized as either acquired or intrinsic, resistance as a single phenotypic feature is usually based on the same mechanisms that are closely related or combined in both of these types. In this review, we summarize the most common changes in retinoid metabolism and action that may affect the sensitivity of a tumor cell to treatment with retinoids. The availability of retinoids can be regulated by alterations in retinol metabolism or in retinoid intracellular transport, by degradation of retinoids or by their efflux from the cell. Retinoid effects on gene expression can be regulated via retinoid receptors or via other molecules in the transcriptional complex. Finally, the role of small-molecular-weight inhibitors of altered cell signaling pathways in overcoming the resistance to retinoids is also suggested.

## 1. Introduction: Primary and Secondary Drug Resistance

As a defective or inactive differentiation program is a typical feature of many human malignancies, treatment promoting cell differentiation represents an important therapeutic strategy. During the past decades, several strategies aiming to overcome this block in cell differentiation and to acquire a non-malignant mature phenotype or to enter the apoptotic pathways were introduced into clinical practice [1]. 

Among others, retinoids as derivatives of vitamin A represent a popular group of differentiation inducers that are successfully used in oncology for treatment of acute promyelocytic leukemia (APL) in adults and of neuroblastoma (NBL) in children. The therapeutic potential of retinoids is based on their key role in regulation of cell differentiation, growth, and apoptosis, which provides a basis for their use both in cancer therapy and in chemoprevention. Nevertheless, patients treated with retinoids often exhibit or develop resistance to this therapy [2]. 

Intrinsic or primary drug resistance refers to a tumor that shows insignificant or no response to the therapy at the onset of treatment [3]. While primary resistance is very rare among newly diagnosed APL and a relapse of APL is often associated with acquired resistance to retinoids [4], evidence suggests that solid tumors can develop intrinsic resistance to retinoids during carcinogenesis [5]. Acquired or secondary drug resistance occurs when a tumor that initially responded to treatment is found to be no longer sensitive to the same therapy [3]. 

Many possible molecular mechanisms of this resistance to retinoid therapy were recently described. Although drug resistance is commonly categorized as either acquired or intrinsic [3], as described above, the mechanisms of resistance are usually similar or the same in both situations, as they are based on natural mechanisms that play a role in protecting tissues from xenobiotic accumulation and from resulting toxicity [3,6]. Different mechanisms of resistance are usually closely related or combined. Therefore, resistance to retinoids can be described as a multifactorial phenomenon.

## 2. Retinoids as Inducers of Cell Differentiation

In mammalian organisms, retinoids are essential for normal embryogenesis and development, for vision, immunity, reproduction, as well as for maintenance of differentiated epithelial tissues. The capability of retinoids to induce cell differentiation of transformed cells was confirmed on various tumor types including promyelocytic leukemia [7], NBL [8], choriocarcinoma [9], and teratocarcinoma [10]. For example, the differentiating effects of retinoids encompass the increased expression of cell surface differentiation markers in myeloid leukemia cells [11], alterations in cell morphology and formation of neurite extensions in NBL [12], and growth inhibition and typical changes in gene expression in NBL and medulloblastoma cells [12,13,14].

**Figure 1:**
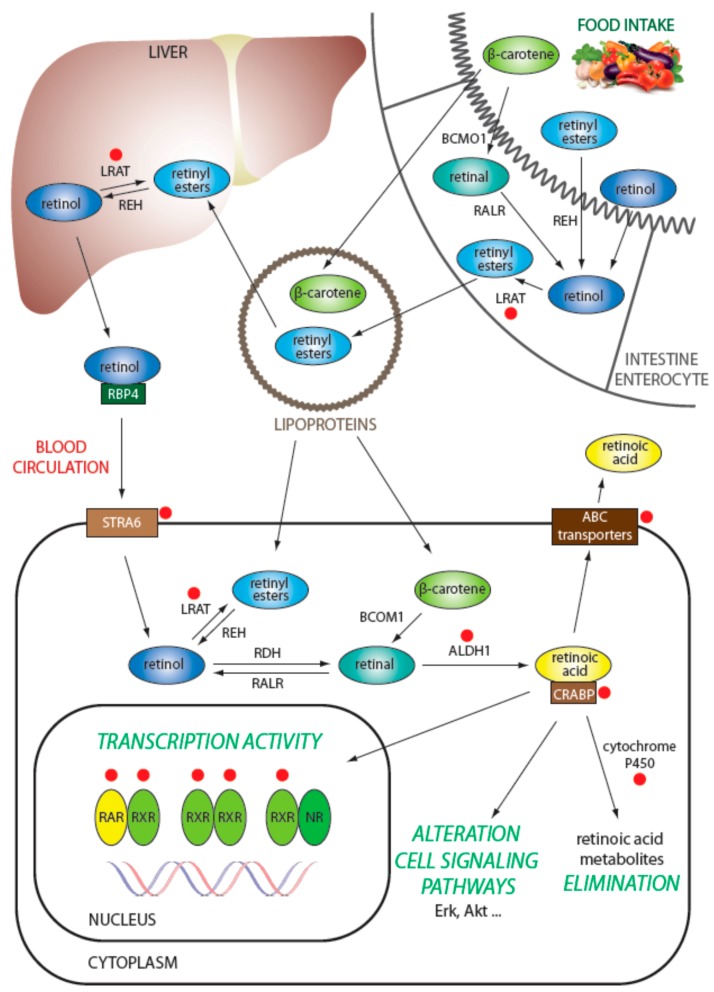
Retinoid transport pathways within an animal organism. Various metabolic forms of retinoids are shown in relation to their localization, transport, and fate. Red dots indicate key players with described role in different mechanisms of resistance to retinoids. LRAT: lecithin-retinol acyltransferase; REH: retinyl ester hydrolase; STRA6: receptors stimulated by retinoic acid 6; ABC: ATP-binding cassette; RALR: retinal reductase; RDH: retinol dehydrogenase; CRABP: cellular retinoic acid binding proteins.

Retinoids are lipophilic isoprenoids composed of a cyclic group and a linear chain with a hydrophilic polar group. Various forms of retinoids are generated through modifications on the polar group of the molecule [15]. For humans, provitamin A serves as a natural source of retinoids, and it is acquired from diet in the form of carotenoids that are metabolized into retinal in the gut. Retinal is then reduced into retinol, which is carried by retinol-binding protein 4 (RBP4) in plasma as a main source of retinoids for the human body. Furthermore, retinoids can also be acquired from diet directly, and they are present in the plasma in lipoproteins, mainly in chylomicrons [16] (Figure 1).

**Figure 2:**
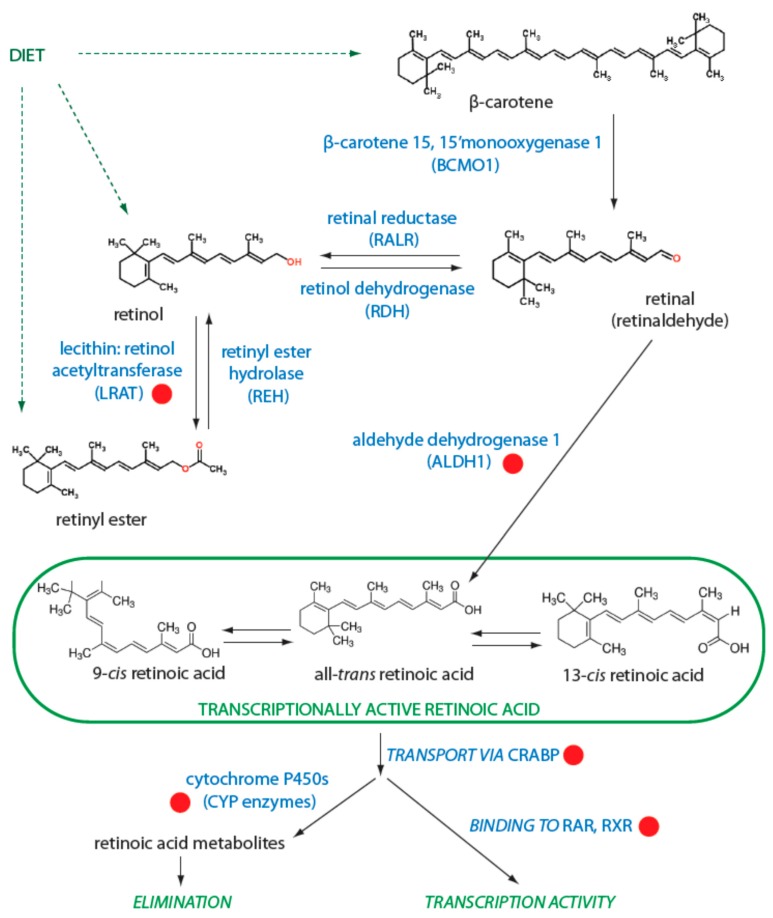
Retinoid metabolism. The involvement of different enzymes in the metabolism of retinoids is shown. Transcriptionally active isomers of retinoic acid are highlighted by a green frame. Red dots indicate key players with described role in different mechanisms of resistance to retinoids. RAR: retinoic acid receptor; RXR: retinoid X receptor.

The liver serves as the main storage site of retinoids in the body, and this organ also accurately regulates the retinol level in plasma. Cellular uptake and efflux of retinol transported by RBP4 occurs via receptors stimulated by retinoic acid 6 (STRA6). Thus, retinol is a transport form and a precursor that is enzymatically activated to retinoic acid via a two-step oxidation process (Figure 2). Retinol can be esterified to retinyl ester by lecithin-retinol acyltransferase (LRAT) or can be oxidized to retinal (also known as retinaldehyde). Retinyl esters can be hydrolyzed back to all-*trans*-retinol by retinyl ester hydrolase (REH) [17]. Similarly, other retinoids acquired directly from diet may be converted to retinal. In humans, retinal is oxidized into retinoic acid by the isoforms of aldehyde dehydrogenase 1 (ALDH1), specifically, ALDH1A1, ALDH1A2, and ALDH1A3 [18]. 

Retinoic acid is present in the organism in several stereoisomeric forms: all-*trans* retinoic acid (ATRA), 13-*cis* retinoic acid (13*cis*RA), and 9-*cis* retinoic acid (9*cis*RA). ATRA can be isomerized through a non-enzymatic process to *9cis*RA or 13*cis*RA isomers [15]; similarly, both 13*cis*RA and 9*cis*RA can be isomerized to ATRA [19,20]. The latter type of isomerization appears to be more common because ATRA was proved to be transcriptionally the most active isomer [15]. The availability of retinoids within cells is closely connected to their transport proteins because retinoids show limited stability and low solubility in aqueous solutions. The main pathway for transport of retinoic acid through the cell and into the nucleus employs cellular retinoic acid binding proteins (CRABP) I and II. CRABP proteins are water soluble, and both CRABP I and II remain in the cytosol in the absence of ligand. When a ligand is present, it is delivered by CRABP proteins directly to the canonical retinoic acid receptor (RAR) or retinoid X receptor (RXR) proteins or to others such as peroxisome proliferator-activated receptor (PPAR) β/δ. Thus, CRABP proteins can modulate the fate of a ligand by transport and binding of the ligand to several different receptors. CRABP proteins can also regulate the amount of retinoids within the cell by delivering ATRA to the catabolic pathway in which it is degraded by cytochrome P450 (CYP) enzymes [21]. Alternatively, the intracellular availability of retinoids can also be regulated by their excretion from the cell by ATP-binding cassette (ABC) transporters.

As we mentioned above, the activity of retinoids is primarily mediated by nuclear receptors, which work as selective ligand-activated transcription factors. 

The canonical receptors are RAR and RXR. Both of them have several isoforms arising from alternative promoters as well as from splicing variants. This variability is related to the diversity and flexibility of functions regulated by vitamin A and its metabolites. In general, RAR can dimerize with RXR, but RXR can act either on its own through an RXR homodimer or with a variety of partners: PPARs, liver X receptors (LXRs), thyroid hormone receptors (THRs), farnesoid X receptor (FXR), etc. [22]. 

Regulation of gene transcription by retinoids is typically caused by constitutive binding of RAR homodimers or RAR-RXR heterodimers to specific retinoic acid response elements (RAREs) found in the regulatory regions of target genes. Similarly, RXR homodimers can bind to retinoid “X” responsive elements (RXREs), and heterodimers of RXR with other nuclear receptors (except RAR) bind various distinct response elements. Via this molecular mechanism, retinoids are involved in the control of many important physiological processes in the organism [23].

**Figure 3:**
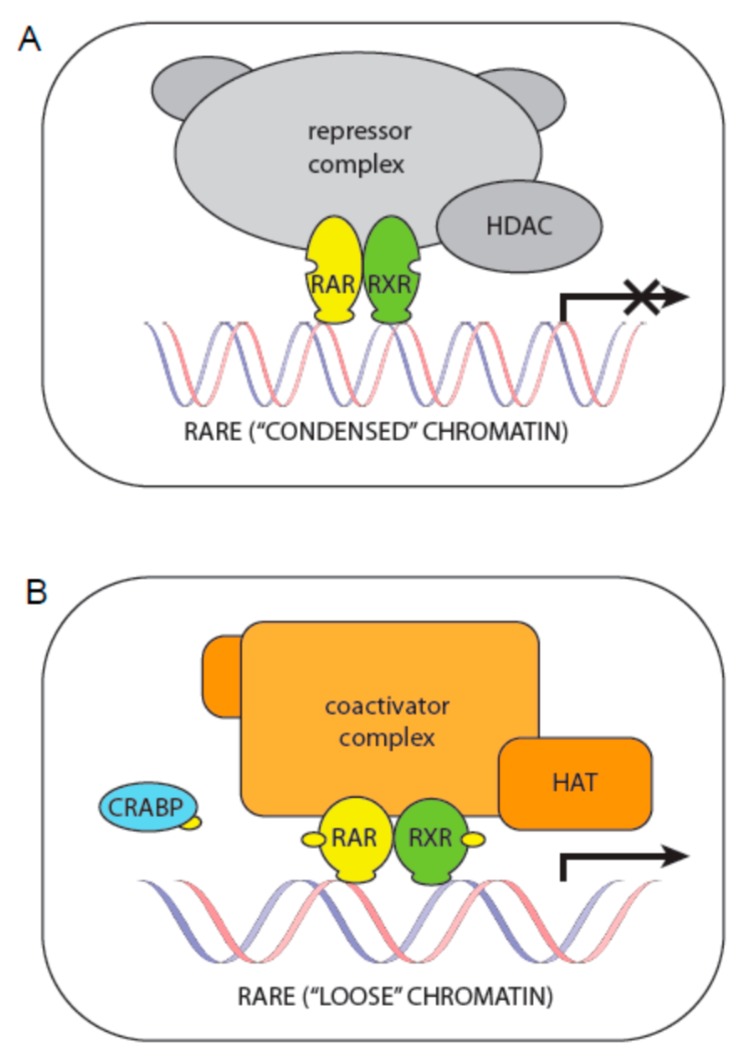
Canonical regulation of transcription by retinoids. (**A**) Without retinoic acid (yellow dots), RAR-RXR receptors together with corepressors form a repressor complex with histone deacetylase activity, which keeps the chromatin in a “condensed” state; (**B**) Addition of retinoic acid—delivered to the nucleus by CRABP—induces conformational changes in RAR:RXR receptors, the repressor complex is released and a coactivator complex with histone acetyltransferase activity is formed. Such activity induces a “loose” state of chromatin required for active gene transcription.

The best described regulation is that by RAR-RXR heterodimers. In the absence of retinoic acid, these heterodimers are associated with corepressors that serve as adaptors recruiting high-molecular-weight complexes endowed with activity of histone deacetylases (HDACs). The removal of acetyl groups from histone tails by HDACs, which induces chromatin condensation and prevents gene transcription, is one of the most important epigenetic mechanisms. The presence of retinoic acid delivered to the nucleus by CRABP induces a conformational change in RAR. Consequently, the corepressor is released, the RXR is free to bind its ligand, and coactivators with histone acetyltransferase activity (HAT) bind to both RXR and RAR. These coactivators also induce a “loose” state of chromatin required for active gene transcription [24] (Figure 3).

However, new methods such as chromatin immunoprecipitation and deep sequencing (ChIP-seq) have provided more complex insight into this mechanism: the treatment with retinoic acid induces widespread changes in RAR binding sites and in partners participating in the assembly of a transcriptionally active RAR-RXR complex. Moreover, these new methods also showed that, in addition to the regulation of transcription within the cell nucleus, RARs also have a number of other extranuclear and non-transcriptional effects such as rapid and transient activation of several kinase cascades [25,26,27,28,29].

## 3. Molecular Mechanisms of Resistance to Retinoids

As obvious from this description of the fate of retinoids and their physiological role within an organism, the resistance to retinoids as a single phenotypic feature can originate from various mechanisms. In the next parts of this section, we describe the most common changes in retinoid metabolism and actions that may affect the sensitivity of a tumor cell to treatment with retinoids.

### 3.1. Regulation of Retinol Metabolism

As retinol is the primary source of retinoids within cells, changes in retinol metabolism can contribute to the development of resistance to retinoids. 

The concentration of retinol in the cytoplasm is primarily regulated by the STRA6, LRAT, and REH enzymes [30]. For example, LRAT knockdown restored sensitivity to retinoids in melanoma cells. In these cells, significant upregulation of the CYP26A1 and STRA6 genes in LRAT knockdown cells was identified in mRNA levels, suggesting their possible involvement in mediating resistance to retinoids [31]. A direct relationship between LRAT expression and regulation of ATRA levels was proved in the B16F10 murine melanoma cell line. These cells are sensitive to retinoids and they have no endogenous expression of LRAT. Furthermore, a modified B16F10 cell line overexpressing LRAT showed a markedly reduced effect after treatment with retinoids [32]. 

A partial explanation for this mechanism of resistance was achieved previously using human keratinocytes. In these cells, LRAT activity is induced by retinoic acid, and such induction increases the esterification of retinol, which concomitantly reduces the oxidation of retinol through retinal to retinoic acid [33]. Nevertheless, from the results described by Amann and colleagues, it is apparent that the mechanism of resistance in transformed cells is the same: via regulation of LRAT activity, retinoic acid can manage its own level by controlling the availability of retinol required for conversion to retinoic acid. 

Similarly, the importance of the regulation of retinoic acid biosynthesis for resistance to retinoids was also described in neuroblastoma cells. As mentioned above, ALDH1 isoforms oxidize retinal to retinoic acid. It was shown that the expression of ALDH1A2 isoform significantly correlates with poor prognosis in neuroblastoma patients and is associated with the resistance of neuroblastoma cells to 13*cis*RA [34]. It was also reported that the exogenous addition of ATRA or other synthetic retinoids can reduce ALDH activity in cancer cells [35]. Suppression of ALDH activity by retinoids is probably a part of the regulation by negative feedback. As increased expression of ALDHs is recognized as one of the hallmarks of cancer stem cells, this mechanism of retinoid action within the cell may explain why retinoids are considered promising substances for overcoming chemoresistance in cancer stem cells [18,36].

### 3.2. Intracellular Transport of Retinoids

Oxidation of retinol to retinoic acid occurs in the cytoplasm near the cell surface. Intracellular transport of ATRA to the cell nucleus is mediated by the CRABP I and CRABP II accessory proteins. This key role of CRABP proteins implies that alterations in their expression may be associated with resistance to retinoids. However, the published findings on this topic are almost contradictory because the same transporting system using CRABP is also involved in the inactivation of retinoic acid by oxidative enzymes belonging to the P450 family [21]. 

Downregulated expression of CRABP II was found in retinoid-resistant medulloblastoma cell lines in comparison with retinoid-sensitive cell lines expressing CRABP II endogenously. Furthermore, sensitization of retinoid-resistant cell lines was described after restoration of CRABP II expression [37].

In contrast, several studies on APL cells reported that CRABP II expression was increased at the time of APL relapse if compared with the CRABP II levels before initiating treatment with ATRA [38,39]. Nevertheless, a later study suggested that the differences in CRABP II expression and its retinoic acid-binding activity do not cause the development of clinically acquired resistance to retinoids in APL patients but rather the constitutive expression of CRABP II could facilitate the response of APL cells to treatment with retinoic acid [40]. Similarly, no alterations in CRABP II expression or its binding activity were found in breast cancer [41] or NBL [42] cell lines. 

Taken together, these data suggest that constitutive expression of CRABP is more important in this mechanism of retinoid resistance rather than a complete lack of CRABP expression. As mentioned above, CRABP transports retinoic acid not only to the receptors to regulate transcription in the cell nucleus but also to locations for degradation. Thus, the regulatory role of CRABPs in the various mechanisms of resistance to retinoids is probably more important than the simple regulation of CRABP-mediated intracellular transport by downregulated expression of CRABPs.

### 3.3. Degradation of Retinoids

The degradation of retinoic acid isomers is one of the mechanisms regulating the concentration and availability of retinoids in specific tissues and cells. This clearance is mediated by enzymes belonging to the cytochrome P450 family. Among them, especially CYP26 enzymes have been reviewed as being important for ATRA degradation. Nevertheless, the detailed mechanism by which CYP26 enzymes participate in the catabolism of 9*cis*RA and 13*cis*RA, or of 4-oxo-RA metabolites, is not known [43].

The metabolically most active isomer is ATRA, and the natural intracellular isomerization of other retinoic acid isomers to ATRA is well known [20]. When ATRA is administered to humans, it is able to induce its own catabolic degradation probably via CYP26. For example, a high metabolic rate resulting from the autoinduction of RA catabolism was found in retinoic acid-sensitive breast cancer cell lines only and not in resistant cell lines [41]. Similarly, relapse and resistance to retinoids was associated with a rapid and marked decrease of retinoid levels in the plasma of APL patients [44,45]. 

As ATRA is predominantly metabolized by CYP26 enzymes, it has been proposed that inhibition of these enzymes will increase the concentrations of ATRA in target tissues; thus, the inhibition of CYP26 enzymes during the therapeutic administration of ATRA or 13*cis*RA could be used to combat the resistance. Based on this hypothesis, several inhibitors known as retinoic acid metabolism-blocking agents (RAMBAs) have been developed and tested [46].

Nevertheless, some researchers warn that resistance to retinoids could result from either local induction of CYP26 expression in cancer cells or from the induction of systemic degradation of ATRA, in which more key players should participate [43]. The main limitation on the targeting of retinoic acid catabolism by inhibitors is the variability of the cytochrome P450 family. Although the degradation of ATRA is mediated predominantly by CYP26A1 and CYP26B1 enzymes, other retinoic acid isomers are also metabolized by CYP3A4 and CYP2C8 enzymes, and these cytochromes probably also play a role in the catabolism of exogenously administered ATRA and 13*cis*RA [43]. Moreover, due to different expression levels of CYP26 enzyme isoforms in various tissues in the organism, the inhibition of all these enzymes in a tissue-specific manner may have different pharmacological consequences. Finally, it is not clear whether inhibition of CYP26A1 and/or CYP26B1 will increase circulating concentrations of ATRA in plasma or if it will subsequently lead to increased concentrations of ATRA in a specific tissue.

### 3.4. Efflux of Retinoids from the Cell

In addition to the intracellular degradation of retinoids, their active efflux from the cell to the extracellular space can contribute to a decrease of retinoid concentrations within the cell. Proteins belonging to the superfamily of ATP-binding cassette (ABC) transmembrane transporters play a key role in this transport. Overexpression of genes encoding ABC transporters is very often associated with multidrug resistance (MDR), i.e., with resistance to a wide range of cytotoxic agents. Three of the ABC transporters were commonly reported to be responsible for MDR in various tumor types: P-glycoprotein (Pgp, ABCB1), multidrug resistance protein 1 (MRP1, ABCC1), and breast cancer resistance protein (BCRP, ABCG2) [47].

Association between MRP1 overexpression and resistance to retinoids was demonstrated in NBL cell lines: those with constitutively overexpressed MRP1 were markedly more resistant to treatment with ATRA [48]. However, the detailed mechanism of this type of resistance is apparently more complex. It was described that MRP1 is a direct transcriptional target of *N*-myc protein, transcription of which is downregulated by retinoids [49]. Thus, the treatment of NBL cell lines with retinoic acid leads to the coordinated downregulation of *N*-myc and MRP1. 

Another study indicates that ATRA can increase Pgp activity in leukemic cells [50]. In contrast, the inhibition of Pgp- and BCRP-mediated substrate transport and the substrate-stimulated ATPase activity of these transporters were described in murine cells treated with 13-*cis*-RA, retinol, and retinyl-acetate, while 9-*cis*-RA and ATRA showed no effect on the activities of these ABC transporters [51].

### 3.5. Regulation of Transcription via Retinoid Receptors

As mentioned above, all biological effects of retinoids within the cell are realized by two types of retinoid nuclear receptors: RAR and RXR. Each of them has three subtypes (α, β, and γ), and each of these subtypes has different isoforms. Alterations in signaling through these nuclear receptors are undoubtedly very important for the development of resistance to retinoids. There are many published studies on the possible relationship between RAR receptors and resistance to retinoids; research focused on the RARα receptor is usually performed on APL cells because a PML–RARα complex is a characteristic feature of the typical APL complex, whereas studies on cell lines derived from solid tumors are mainly aimed at the RARβ receptor. 

Interestingly, the expression of RARβ is frequently decreased or completely downregulated in primary solid tumors and in their metastasis compared to adjacent non-tumor tissue [52,53,54]. It was described that cells in which RARβ is deleted or its expression is impaired are selected in the cell population during the phase of rapid tumor growth [55]. Epigenetic silencing of RARβ2 was reported to induce resistance to retinoids in breast and prostate cancer cell lines [56]. Similarly, differences in sensitivity to retinoids caused by alterations in histone H3 acetylation in the *RARB* promoter were described in thyroid carcinoma cell lines [57]. 

The importance of RARβ in the development of retinoid resistance was also shown in several studies on NBL cells. Increased sensitivity to retinoids was identified in NBL cell lines transfected with a vector expressing RARα/RARβ/RARγ: whereas transfectants overexpressing RARβ demonstrated marked growth inhibition without any morphological evidence of differentiation, RARα transfectants showed similar sensitivity to retinoids as control cells. RARγ transfectants demonstrated resistance to neuritogenesis but not to the growth inhibition induced by retinoids [58].

Although changes in expression of RAR and RXR receptors are typical for various tumor types [53,59], the relationship between these alterations and resistance to retinoids is still unclear. Despite several papers that mentioned a special role of RXRα receptor in resistance mechanisms, maximum resistance to retinoic acid was shown in ovarian cancer cell lines with downregulation of both RARα and RXRα in contrast to cell lines with reduced levels of either RARα alone or RXRα alone [60].

In addition to the alterations in RAR and RXR receptor expression, special attention is paid to the role of phosphorylation in the regulation of RAR functions. In breast cancer cell lines, it was shown that deregulation of cytoplasmic signaling cascades ending at Akt kinase or at other MAP kinases (Erk, JNK, p38MAPK, for example) can lead to the aberrant phosphorylation of RAR receptors. Subsequently, RARα is degraded and/or its transcriptional activity is minimized. Both these effects can then cause resistance to the antiproliferative action of retinoids [53,61].

Mutations in the ligand-binding domain of the RAR receptor represent another possible way that the receptor function can be deregulated. Such mutations responsible for resistance to retinoids are well described in the RARα region of a *PML-RARA* fusion gene in APL cells [62,63,64]. This mutant PML-RARα protein causes various alterations in binding both to ligand and to nuclear coregulators of transcription. Consequently, all these changes lead to the various levels of inhibition of retinoid-induced transcription [65,66]. The results achieved on non-leukemia cells are more rare and more inconsistent. Truncated RARα caused resistance to retinoids in an embryonal carcinoma cell line [67] whereas epithelial cells with different types of truncated RARα mutants were more sensitive to treatment with retinoic acid [68]. In breast cancer cell lines, inhibition of endogenous RARα expression led to growth stimulation through a non-RAR-mediated signaling pathway [69].

### 3.6. Regulation of Transcription via Other Molecules in the Transcriptional Complex

Cancer cells may downregulate RARβ expression by other mechanisms such as the loss of coactivators or overexpression of a corepressor. One of the promising components of the RAR corepressor complex is xeroderma pigmentosum group A (XPA)-binding protein 2 (XAB2). This protein is involved in pre-mRNA splicing, transcription, and transcription-coupled DNA repair. It was shown that XAB2 is associated with RARα and histone deacetylase 3 in cell nuclei, and overexpression of XAB2 inhibits ATRA-induced cell differentiation in a human rhabdomyosarcoma cell line. In contrast, the knockdown of XAB2 using siRNA increased ATRA-induced cell differentiation of the HL60 human promyelocytic leukemia cell line. Finally, the ATRA-resistant IMR-32 NBL cell line was able to undergo cell differentiation induced by ATRA after the same knockdown of XAB2 using siRNA [70]. 

ZNF423 functioning as a cofactor for RARα/RXRα transactivation is another key player in the regulation of transcription. It was demonstrated that suppression of ZNF423 leads to increased proliferation activity and to resistance to RA-induced cell differentiation in NBL cell lines, whereas ZNF423 overexpression caused inhibition of proliferation and enhanced cell differentiation [25]. 

In the absence of a ligand, RAR/RXR actively represses transcription through association with the corepressor complex and recruitment of histone deacetylases (HDAC) that prevent chromatin opening (Figure 3). Increased HDAC activity is a common causal factor in human cancers that leads to the transcriptional silencing of tumor suppressor genes and that can also reduce the activity of retinoids. HDAC inhibitors are able to block these activities and thus promote transcription. In particular, the combination of HDAC inhibitors with other anti-neoplastic agents such as retinoids seems to be very promising [71]. Nevertheless, the application of these epigenetic drugs is associated with some risks. For example, the combination of ATRA with an HDAC inhibitor led to an unexpected opposite effect: increased aggressiveness of glioma xenografts [72]. Thus, the use of HDAC inhibitors in combination with retinoids in clinical practice is still questionable.

## 4. Conclusions

Although resistance to retinoids is a known phenomenon with important therapeutic consequences for many years, the roots of this are apparently heterogeneous in various patients. In addition to the main mechanisms of resistance as described above, little is known about downstream regulation of retinoid action within the cell. In particular, the interplay of signaling pathways and possible use of small-molecular-weight inhibitors to overcome the resistance to retinoids seems to be a very promising strategy for patients suffering with APL or NBL. For example, such an effect on retinoid-resistant leukemia cells was achieved with MEK inhibitor or Src-family kinase inhibitors under in vitro conditions [73,74]. Similarly, a hyperactive RAS signaling pathway was recently shown in retinoid-resistant NBL cells with recovery effects from inhibition of the MEK-ERK component of the MAPK signaling network [75]. In any case, seeking molecules or rather biomarkers that may indicate possible resistance to retinoids in an individual patient seems to be of high importance.

## Abbreviations

ABC	ATP-binding cassette
ALDH	aldehyde dehydrogenase
APL	acute promyelocytic leukemia
ATRA	all-*trans* retinoic acid
BCRP	breast cancer resistance protein
CRABP	cellular retinoic acid binding protein
CYP	cytochrome P450
FXR	farnesoid X receptor
HAT	histone acetyltransferase
HDAC	histone deacetylase
LRAT	lecithin-retinol acyltransferase
LXR	liver X receptor
MDR	multidrug resistance
NBL	neuroblastoma
Pgp	P-glycoprotein
PPAR	peroxisome proliferator-activated receptor
RAR	retinoic acid receptor
RARE	retinoic acid response element
RBP4	retinol-binding protein
REH	retinyl ester hydrolase
RXR	retinoid X receptor
RXRE	retinoid X response element
STRA	receptors stimulated by retinoic acid
THR	thyroid hormone receptor
XAB2	xeroderma pigmentosum group A-binding protein 2
9*cis*RA	9-*cis* retinoic acid
13*cis*RA	13-*cis* retinoic acid
